# Animal fluency in people with Parkinson's disease: Item‐based performance before and after deep brain stimulation surgery

**DOI:** 10.1111/jnp.70026

**Published:** 2025-12-08

**Authors:** Adrià Rofes, Nikki Janssen, Janine Rook, Eva de Ronde, R. Saman Vinke, Rianne A. J. Esselink, Annelien A. Duits

**Affiliations:** ^1^ Center for Language and Cognition Groningen (CLCG) University of Groningen Groningen The Netherlands; ^2^ Research School of Behavioural and Cognitive Neurosciences University of Groningen Groningen The Netherlands; ^3^ Department of Medical Psychology Radboud University Medical Center Nijmegen The Netherlands; ^4^ Department of Psychiatry Radboud University Medical Center Nijmegen The Netherlands; ^5^ Department of Neurosurgery Radboud University Medical Center, Donders Institute for Brain, Cognition and Behaviour Nijmegen The Netherlands; ^6^ Department of Neurology Radboud University Medical Center, Donders Institute for Brain, Cognition and Behaviour Nijmegen The Netherlands; ^7^ Department of Medical Psychology Maastricht University Medical Center Maastricht The Netherlands

**Keywords:** clusters, DBS, executive function, fluency, Parkinson, switches, word properties

## Abstract

People with Parkinson disease (PD) after surgery for deep brain stimulation (DBS) of the subthalamic nucleus (STN‐DBS) often decline in animal fluency due to impairments in executive functions and/or language. Item‐based measures of animal fluency may shed light on the specific nature of this decline, and into the strategies used when performing this task. We aimed to investigate the mechanisms of decline in animal fluency by revealing impairments in language and/or executive functions in people with PD before and after STN‐DBS by using item‐based characteristics, the total number of correct words, average cluster size, number of switches and scores on tests for language and executive functions. People with PD (*N* = 61) produced fewer words and switches than healthy controls (*N* = 40) before and after STN‐DBS surgery. After surgery they additionally produced smaller clusters and shorter words than healthy controls. Comparing pre‐ and post‐surgery, people with PD produced fewer words, fewer switches, smaller clusters, more frequent and earlier‐acquired words after surgery. Average cluster size predicted total number of words before surgery. No item‐based measures predicted total number of words after surgery. Average cluster size before surgery correlated with object naming, not with executive functions. Item‐based measures indicated difficulties in executive functions and language processing. New to the literature, the correlation of cluster size with object naming may stress difficulties in lexical retrieval before surgery. Finding no item‐based measures predicting the total number of words after surgery may indicate a different type of impairment not accounted for in our analyses. Replication is needed.

## INTRODUCTION

Verbal fluency tasks are part‐and‐parcel of neuropsychological assessments of people with neurodegenerative disorders, as they are rapid to administer and provide a reliable measure of language and executive functions (Arroyo‐Anlló et al., 2011; Marczinski & Kertesz, 2006; Ramos & Machado, 2024; Rofes et al., 2019, 2020). Animal fluency is a type of verbal fluency task that engages the lexico‐semantic system to retrieve as many words (i.e., names of animals) as possible in 1 or 2 min. Also, it engages executive functions such as inhibition, updating and monitoring to adhere to the task‐rules such as only producing animals, avoiding repetitions or not producing proper nouns (Fong et al., 2020; Gordon et al., 2018; Oh et al., 2019; Rofes et al., 2023; Shao et al., 2014; Whiteside et al., 2016).

Analyses of animal fluency traditionally rely on counting the total number of correct words produced (Bousfield & Sedgewick, 1944; Henry & Crawford, 2004; Rosen, 1980). These analyses have been supplemented with counting how words group in semantic clusters (e.g., farm animals, African animals) and how people switch from one cluster of semantically‐similar words to another cluster (e.g., Abwender et al., 2001; Troyer et al., 1997). The literature has seen a recent advent of studies looking at item‐based characteristics of the words produced, such as the frequency (in a corpus) of the words used, when people acquired the words, and their length in graphemes (De Marco et al., 2023, 2025; Rofes et al., 2020; Vonk et al., 2019). These studies are complementary to other work where total number of correct words in fluency tasks, as well as average cluster size and number of switches, have been correlated with scores on neuropsychological tests (e.g., digit span, matrix reasoning) or variables extracted from these tests, such as lexical retrieval speed in object naming (Fong et al., 2020; Gordon et al., 2018; Shao et al., 2014).

Item‐based analyses are not new in cognitive neuropsychology and also not in fluency tests: studying word properties can help elucidate the nature of the impairments that people may have, for example, whether the impairments reflect issues with the representation of concepts versus difficulties accessing lexical items (Marczinski & Kertesz, 2006; Shallice et al., 2000; Whitworth et al., 2014). Learning about these differences is relevant to better understand the impairments that people face; which strategies they use to produce as many words as possible in a short period of time; and what cognitive factors drive differences in performance in the case of neurodegenerative disease, before and after a behavioural, surgical and/or pharmacological procedure.

People with Parkinson disease (PD) produce fewer words in animal fluency relative to aged‐matched controls (Henry & Crawford, 2004; Parsons et al., 2006; Tagini et al., 2021; Wyman‐Chick, 2016). Studies of clusters and switches indicate that people with PD produce fewer switches than healthy controls (Galtier et al., 2017; Troyer et al., 1998). Relative to healthy controls, people with PD produce more words acquired earlier in life in animal fluency (Wagner et al., 2020). Differences in the frequency or the typicality of words have not been found in animal fluency performance between both groups (Herrera et al., 2012; Zabberoni et al., 2017). However, healthy controls produce words with lower frequency than people with PD in letter fluency (Foster et al., 2008), vegetable fluency (Tiedt et al., 2022) and in action fluency (Herrera et al., 2012).

People with PD also have difficulties in tasks other than verbal fluency, such as picture naming (actions more than objects, cf. Longo et al., 2025), producing verbs in the past tense (i.e., ‘worked’ for ‘to work’), and completing sentences with verbs (Anyfantis et al., 2024; Camerino et al., 2024; Colman et al., 2009; Verhaegen et al., 2022). Some of these difficulties have been reported in other populations with damage in the basal ganglia, such as subcortical stroke, small vessel disease and Huntington's disease (Camerino et al., 2024). Finally, people with PD have difficulties during the production of narratives and spontaneous language (D'Ascanio et al., 2023; Lowit et al., 2022).

People in the advanced stage of PD may benefit from deep brain stimulation (DBS) surgery—a procedure where electrodes are usually placed bilaterally in the subthalamic nucleus (STN), and less commonly unilaterally or in the globus pallidus internus (GPi) or ventral intermediate nucleus (VIM) of the thalamus (Benabid et al., 2001, 2009; Kübler‐Weller et al., 2025; Longo et al., 2025). Fluency tasks are commonly used before and after deep brain stimulation of the subthalamic nucleus (STN‐DBS), along with other language‐related tests that are less often used such as picture naming (Busteed et al., 2025; Vos et al., 2021). Whereas there is inconclusive evidence for decline in measures for memory, executive function and processing speed, post‐operative decline in verbal fluency has been consistently reported, with preliminary evidence for larger decline in semantic than letter fluency (Tröster, 2024). Compared to people with PD and best medical treatment, decline in verbal fluency is higher in those undergoing surgery for (bilateral) STN‐DBS (Jahanshahi et al., 2022). People with PD treated with STN‐DBS produce fewer words in fluency tests than those with only medication (John et al., 2021; Longo et al., 2025; Mulders et al., 2021; Vos et al., 2021; Wyman‐Chick, 2016). There are no differences in the total number of words when DBS is ON or OFF, though the number of switches is higher when DBS is ON than when it is OFF (Vonberg et al., 2016). Also, more words and larger average cluster sizes have been reported in people with low‐frequency STN‐DBS compared to high‐frequency STN‐DBS (Busteed et al., 2025). Difficulties with fluency tests have been related to low scores in functional independence measures (Contarino et al., 2007; Muslimovic et al., 2008). Also, difficulties with communication negatively affect quality of life in people with PD, even 5 years after surgery (Jost et al., 2024).

It has been argued that people after STN‐DBS have problems in executive functions as well as some difficulties with language (Bucur & Papagno, 2023; Klostermann et al., 2022). One study indicated that, after STN‐DBS, people have difficulties with tests that require lexico‐semantic processing, such as difficulties with words that have multiple meanings, identifying semantically unrelated words in a list, generate synonyms, and identify semantic incongruities (Whelan et al., 2003). There are also studies indicating that people with PD after STN‐DBS produce shorter sentences and sentences with more morphosyntactic errors than healthy controls during spontaneous speech tasks (Batens et al., 2014, 2015). Some of the aforementioned results can be modulated by cognitive status, medication dosage, disease duration, motor symptoms, among others (Kübler‐Weller et al., 2025; Tröster, 2024).

In this paper, we performed analyses of animal fluency with a particular focus on item‐based characteristics. These analyses are aimed at elucidating impairments in language and/or executive functions in people with PD before and after STN‐DBS. This is relevant from a theoretical perspective given that, specific to animal fluency, it is unclear whether people with PD before and after STN‐DBS surgery differ from healthy controls regarding item‐based measures of fluency tasks (e.g., frequency, age of acquisition, concreteness, word length, cluster size, number of switches). Also, it seems worth assessing whether (and if so, how) item‐based measures indicate cognitive difficulties relating to executive functions or language. From a more clinical perspective, this work is relevant, as it allows us to get a better understanding of task that is already commonly administered. Hence, opening the door to new and more detailed neuropsychological assessments.

### Aim, objectives and predictions

The overarching aim of this paper is to investigate the mechanisms of decline in animal fluency by assessing impairments in language and/or executive functions in people with PD before and after STN‐DBS by using item‐based characteristics. To do so we (1) compare whether people with PD (before and after STN‐DBS) and healthy controls differ in the total number of words produced in animal fluency and in item‐based factors extracted from correctly produced words (e.g., word frequency, concreteness, number of switches, average cluster size); (2) compare whether people with PD before and after STN‐DBS differ in total word count and item‐based factors; (3) assess which factors predict total word count in animal fluency in people with PD (before and after STN‐DBS) differently than in healthy controls, and (4) examine whether the factors that predict total word count in animal fluency relate to executive functions and/or language as measured with other neuropsychological tests.

For objective (1) we hypothesise people with PD, both before and after surgery, to produce fewer words and have a lower number of switches than healthy controls. The direction for effects of average cluster size and of word properties is difficult to predict, given the sparse number of studies looking at these values in fluency tasks. However, based on literature, we expect individuals with PD to produce words that are ‘easier’ than those of healthy controls. This should result in people with PD producing words that are more frequent, more concrete, shorter, with more phonological/orthographic neighbours, and acquired earlier in life compared to healthy people. For objective (2) we hypothesise people with PD to perform worse after surgery compared to pre‐operatively. The item‐based effects may resemble those of aim (1). In particular, after surgery people with PD may produce fewer switches and possibly clusters of smaller average size, along with words that are more frequent, more concrete, with more phonological/orthographic neighbours, learned earlier in life, and shorter. Regarding objective (3) we expect factors such as number of switches, average cluster size and word frequency, to predict the total word count in both people with PD and healthy controls. Given the sparsity of previous literature, it is hard to predict specific effects for specific item‐based measures of fluency in people with PD. Finally, regarding objective (4) total number of correct words, number of switches and cluster size can correlate with tests measuring executive functions and language. That said, it is hard to make specific predictions due to the paucity of studies in people with PD.

## METHODS

### Participants

A total sample of 101 Dutch‐speaking people participated in the study. The sample included 61 people with PD and 40 healthy controls. The PD group included 27 females ranging in age from 37 to 82 years (pre‐operative mean age = 63 years, SD = 8; post‐operative mean age = 64 years, SD = 8). People with PD ranged in education from 3 (primary school completed and less than 2 years of further education) to 7 (university degree) (mean education = 5.52, SD = 1.07), as calculated with the Dutch Verhage scale (Verhage, 1964). All patients underwent surgery (asleep) for bilateral STN‐DBS at the Radboud University Medical Center, Nijmegen, The Netherlands. Data of pre‐ and post‐operative neuropsychological assessment of 110 patients were collected between November 2018 and December 2023. Those with incomplete data (e.g., incomplete scoring of the animals mentioned, pre‐ and post‐operatively) or not performing animal fluency in Dutch were excluded. People with PD were neuropsychologically assessed in the ‘ON’ condition, prior to and 1 year after surgery. Post‐operatively, the PD group was assessed with medication and on stimulation. Table 1 includes demographics and information regarding cognitive status (Montreal Cognitive Assessment scores), motor symptoms (UPDRS‐III before and after STN‐DBS), levodopa equivalent daily dose of medication (before and after STN‐DBS), body side of symptom onset, and disease duration in months at the time of operation.

**TABLE 1:** Demographic and motor characteristics of PD group.

Sex	Education	Onset_Side*	Duration	MoCA_Pre	MoCA_Post	UPDRS_III_Pre	UPDRS_III_Post	LEDD_Pre	LEDD_Post
26F	5.52 (1.07)	27R	122 (40)	26.1 (2.3)	25.7 (2.7)	15.7 (10.8)	20.5 (8.9)	854 (501)	781 (559)

Abbreviations: Duration, disease duration in months at time of operation; Education, education in years; LEDD, Levodopa Equivalent Daily Dose (Pre = Before STN‐DBS, Post = After STN‐DBS); MoCA, Montreal Cognitive Assessment score (Pre = Before STN‐DBS, Post = After STN‐DBS); Onset_Side, body side of symptom onset (*Unknown for three people); UPDRS III, Unified Parkinson's Disease Rating Scale Part III (Pre = Before STN‐DBS, Post = After STN‐DBS).

The healthy group consisted of 40 Dutch‐speaking people without known brain disease or neurological damage. This group comprised 25 females ranging in age from 47 to 83 years (mean age = 60 years, SD = 8) and in education from 2 to 7 (Verhage, 1964) (mean education = 5.62, SD = 1.19). The healthy group did not differ in terms of age (*t*_(85.41)_ = −1.75, *p* = .08) or education ($\chi_{\left(5\right)}^{2}$ = 3.96, *p* = .55) from the PD group.

All participants signed an informed consent, agreeing to share their data for research purposes. People with PD received no compensation to participate in the study. Healthy participants received around 40€ to participate. Ethics approval for patients was waived by the CMO Radboud UMC (METC‐nr 2023‐16699) and separately obtained for healthy people (CETO 91244583, University of Groningen, Faculty of Arts). The research data and the signed informed consent forms for the PD dataset are securely stored in the Radboud UMC Deep Brain Stimulation database. The research data and informed consent forms for the healthy dataset are securely stored in the University of Groningen drive. All data are pseudonymised and only Radboud UMC staff or University of Groningen staff can grant access to the respective data.

### Materials and procedure

All included participants completed animal fluency. They were given 60 s (as measured with a stopwatch) to name as many words as possible in the category of animals. Participants received no assistance during the task. However, when a participant was hesitant or uncertain the examiner could encourage the participant to produce more words with phrases like ‘you still have some time left’ or ‘maybe try to name a few more’.

The PD group was neuropsychologically assessed with a standard test battery during the pre‐ and post‐operative routine assessment. For the purposes of this study, we focused on the following measures of executive function: cognitive flexibility (Trial Making Test Part B/A; Lezak et al., 2004), inhibition (Stroop card III/II; Stroop, 1935), working memory (Digit span backward WAIS‐IV; Wechsler, 2008); and on the following measure of language: Boston Naming test (Kaplan et al., 1983).

All participants were assessed in a quiet room. Data collection for the PD group was obtained by a trained psychological assistant or master's student. Data collection for the healthy group was obtained by master's students and one of the authors (J.R.).

### Data pre‐processing and scoring of animal fluency

Digital databases with individual score sheets were created. The words were then checked for correctness, given the following criteria: words that belong to categories other than animals (e.g., *tafel*, table) or being part of non‐existent/fantasy creatures (e.g., *eenhoorn*, unicorn) were marked as incorrect; repetitions, foreign words and non‐existing words were also marked as incorrect; general animal categories, like ‘*vogel*’ (bird), ‘*vis*’ (fish) and ‘*insect*’ (insect), were classified as correct. Doubts regarding the spelling of words were checked with a dictionary (van Dale, n.d.).

#### Average cluster size and number of cluster switches

Cluster size refers to the number of responses that fall within a specific subcategory (e.g., ‘pets’). For instance, in the sequence ‘hamster, cat, dog, wolf, coyote, zebra’, the words ‘hamster, cat, dog’ form a cluster of size three because they are all ‘pets’, while ‘wolf’ does not belong to that cluster. The average cluster size is a measure of the typical size of clusters produced by the participant during a particular task. A ‘switch’ occurs when the participant provides a response that does not fit into the previous cluster (i.e., when ‘wolf’ was produced in the previous sequence). The average cluster size and the number of cluster switches were determined using a programme called Semantic Network and Fluency Utility (SNAFU, Zemla et al., 2020). SNAFU identifies the correct words within a fluency task by using specific categorisation schemes which are pre‐determined based on previous research (Rofes et al., 2023; Troyer et al., 1997). A computer programme therefore calculates switches and clusters in the same way for all participants. In this study, the master students transcribed the animals that people produced to count the total number of words. To measure clusters and switches, we used a Dutch version of the animal categorisation scheme from the original SNAFU publication (Zemla et al., 2020). This scheme contains 33 categories (e.g., African, fish, pets, reptiles) and is freely available on‐line for download (github.com/jmjvonk/2022_Rofes_SMART‐MR).

#### Word properties

The following five properties for each of the correct words were extracted: word frequency was extracted from SUBTLEX‐NL, a corpus comprising over 43 million words from Dutch television and film subtitles (Keuleers et al., 2010). Concreteness and age of acquisition were obtained from a comprehensive database of 30,000 Dutch words, where participants provided 5‐point scale concreteness ratings and indicated the age at which they learned each word (Brysbaert et al., 2014). Phonological and orthographic neighbourhood density for each correct word was calculated using CLEARPOND (Marian et al., 2012). This measure considers words differing by one grapheme or phoneme through addition, deletion, or substitution. Word length was determined by counting the number of graphemes in each correct word. To measure word length, we used the function = LEN in Excel.

To maximise the number of values extracted for word properties we performed the following pre‐processing steps to the correct words originally produced: diminutive suffixes were removed (e.g., ‘*hondje*’ becomes ‘*hond*’ (dog), ‘*roodborstje*’ becomes ‘*roodborst*’ (robin)) unless doing so altered the meaning of the word or the diminutive suffix was part of the animal's name (e.g., ‘*stokstaartje*’ (meerkat) or ‘*zilvervisje*’ (silverfish)). Additionally, words in plural form were changed to their singular form (e.g., ‘*muggen*’ (mosquitoes) becomes ‘*mug*’ (mosquito)). Masculine or feminine words were not modified (e.g., ‘*leeuwin*’ remains ‘*leeuwin*’ (lioness)). These criteria were not applied to count the number of graphemes in each correct word, as doing so would alter the original length of the words produced.

When pre‐processing did not help us obtain new values, we imputed the word properties by calculating the mean score per participant for each word property. The number and percentage of imputed values for each word property were no greater than 20% for any of the word properties. Specifically, the missing values per word property in the patient dataset were the following: concreteness (199/2610, 7.6% missing values), frequency (119/2610, 7.3% missing values), age of acquisition (117, 2610, 4.5% missing values), phonological and orthographic neighbourhood (351/2610; 13.4% missing values), word length in graphemes (0% missing values); and the missing values per word property of the healthy dataset were as follows: concreteness (57/1053, 5.4% missing values), frequency (45/1053, 4.3% missing values), age of acquisition (47/1053, 4.5% missing values), phonological neighbourhood (138/1053; 13.1% missing values) and word length in graphemes (0% missing values).

### Analyses

Statistical analyses were performed with R version 4.4.3 (R Core Team, 2024), using the lme4 package for mixed‐effects models (Batens et al., 2015).

To assess differences between total number of words and item‐based measures of animal fluency in people with PD and healthy controls before and after DBS surgery (Objective 1) we performed separate mixed‐effects models, including a fixed effect of group (healthy vs. patient) and random effect of participant, for total number of words and each item‐based variable. We controlled for the following covariates in the PD group: cognitive status (MoCA scores before and after STN‐DBS), motor symptoms (UPDRS‐III before and after STN‐DBS), levodopa equivalent daily dose of medication (before and after STN‐DBS), body side of symptom onset, and disease duration in months at time of operation (i.e., lmer(word_frequency~group + Cognitive_status + Motor_symptoms + Medication + Onset_side + Disease_duration + (1|participant), data = data_preop)). We applied false discovery rate (FDR) correction to the *p*‐values (Benjamini–Hochberg method) to account for multiple comparisons.

Performing a single model with pre‐operative data of the PD group versus controls and a single model with the post‐operative data of the PD group versus controls was not possible because the models did not converge. This is possibly because the single model contains many variables. Also, some of the variables we used are collinear (i.e., mean word frequency was negatively correlated with mean age of acquisition, *r*(99) = −.85, *p* < .001; and sum word count showed a positive correlation with number of switches, *r*(99) = .62, *p* < .001). A possibility to still use a single model is reducing the number of variables with a principal component analysis (e.g., create a single variable for frequency and age of acquisition). However, models with reduced dimensions are more difficult to interpret and, with our dataset, such a model did not converge either.

To assess whether people with PD differ in total word count and item‐based factors before and after DBS surgery (Objective 2), we also performed separate mixed‐effects models including a fixed effect of time (before, after DBS surgery), control for cognitive status, motor symptoms, medication, body side of symptom onset, and disease duration covariates, and a random effect of participant for total number of words and each item‐based variable (e.g., [i.e., lmer(word_frequency~time + Cognitive_status + Motor_symptoms + Medication + Onset_side + Disease_duration + (1|participant), data = data_patients)]). FDR correction for multiple comparisons was applied. These analyses were preferred over using a single model for the same reasons as above.

To understand which factors predict total word count in animal fluency for people with PD different than for healthy controls before and after DBS surgery (Objective 3) we ran separate mixed effects models including an interaction between group and each item‐based measure, and also a random effect of participant (e.g., lmer(word_count~group*word frequency + (1 | participant), data = data_preop)). Here we also controlled for the same covariates (i.e., cognitive status, motor symptoms, medication, body side of symptom onset and disease duration). FDR correction for multiple comparisons was applied. This approach was also preferred to using two single models (pre‐operative, post‐operative data) for the reasons mentioned above.

Finally, to understand whether the factors that predict total word count in animal fluency relate to executive function and language as measured with other neuropsychological tests (Objective 4), we used correlations (i.e., Pearson) between the significant value(s) in the results of Objective 3 and the scores of neuropsychological tests performed in people with PD either before or after surgery. Corrections form multiple comparisons (Bonferroni) were applied.

## RESULTS

The raw data can be accessed upon request. The scripts we used to analyse the data are available here https://osf.io/h2jtg. In Table 2 we present the descriptive data for the total number of words, word properties, switches and clusters for the healthy group, and for people with PD before and after DBS surgery. In Table 3 we present the descriptive data for the neuropsychological tests administered to people with PD before and after DBS surgery.

**TABLE 2:** Descriptive statistics (mean and standard deviation): healthy people and people with PD before and after DBS surgery.

Task	Word count	Concreteness	Frequency	AoA	Orth.Neigh	Phon.Neigh	Length	*N*.Switches	Cluster size
Healthy	26 (7)	4.83 (.04)	2.38 (.17)	6.02 (.47)	7.07 (1.5)	8.77 (2.30)	5.81 (.70)	11 (2.80)	2.22 (.57)
PD‐Pre	22 (5)	4.83 (.06)	2.35 (.22)	6.12 (.55)	7.41 (1.4)	9.11 (1.94)	5.57 (.57)	9 (2.91)	2.38 (.86)
PD‐Post	20 (5)	4.83 (.06)	2.43 (.18)	5.93 (.47)	7.69 (1.4)	9.46 (1.95)	5.42 (.54)	6 (2.52)	1.81 (.44)

Abbreviations: AoA, age of acquisition; length, length in graphemes; cluster size, average cluster size (static); *N*.Switches, number of cluster switches (static); Orth.Neigh, orthographic neighbourhood; PD‐Post, post‐operative assessment, people with Parkinson's disease; PD‐Pre, pre‐operative assessment, people with Parkinson's disease; Phon.Neigh, phonological neighbourhood; Word count, number of correct words.

**TABLE 3:** Descriptive statistics (mean and standard deviation): neuropsychological tests.

Test	TMT B/A	Stroop III/II	Digit Bwd	BNT
PD‐Pre	2.9 (1.3); range = 1.3–7.7	1.63 (.3); range = 1.2–2.6	7.4 (1.6); range = 4–11	160 (11); range = 118–172
PD‐Post	2.9 (1.2); range = 2.3–7.8	1.71 (.05); range = 1.7–4.9	NA; range = NA	159 (13); range = 103–174

Abbreviations: BNT, Boston Naming test BBT total score; Kaplan et al. (1983); Digit Bwd, digit span backward, WAIS‐IV; Wechsler (2008); PD‐Post, post‐operative assessment, people with Parkinson's disease; PD‐Pre, pre‐operative assessment, people with Parkinson's disease; Stroop III/II, Stroop card III/II; Stroop (1935); TMT B/A = Trial Making Test Part B/A; Lezak et al. (2004).

### PD versus healthy controls (objective 1)

**FIGURE 1:**
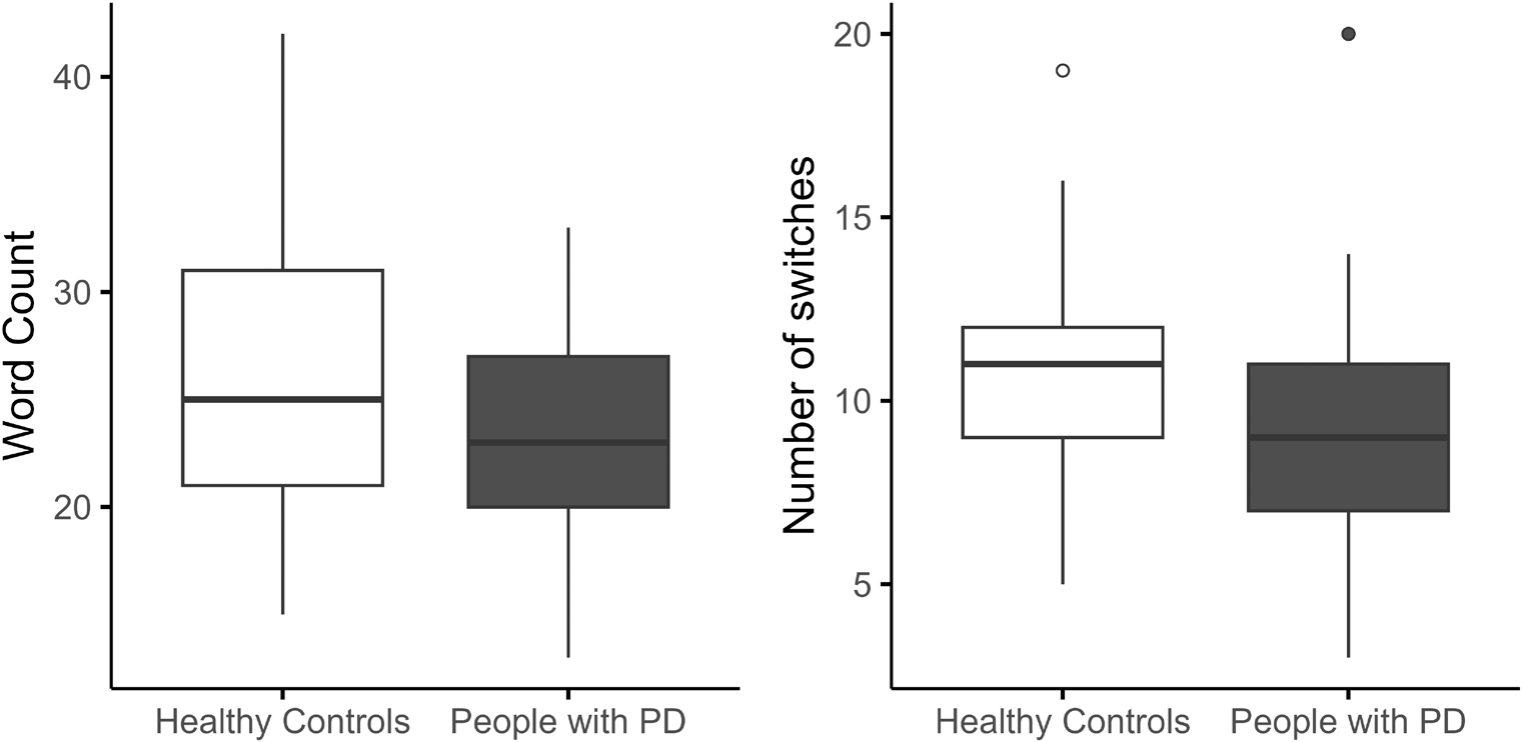
Significant differences between PD and healthy controls before DBS surgery. DBS, deep brain stimulation; PD, Parkinson's disease.

Before surgery, people with PD produced significantly fewer words than healthy controls (β = −3.24, SE = 1.02, *t* = −3.16, adjusted *p* = .047; see Figure 1). This effect was moderate in size (Cohen's *d* = −.45). Similarly, patients made significantly fewer switches than healthy controls (β = −1.65, SE = .55, *t* = −3.03, adjusted *p* = .0495; Cohen's *d* = −.43). No significant differences were observed between groups in average cluster size (adjusted *p* = .51), mean age of acquisition (adjusted *p* = .55), mean concreteness (adjusted *p* = .65), orthographic neighbourhood density (adjusted *p* = .51), phonological neighbourhood density (adjusted *p* = .51) or word frequency (adjusted *p* = .65). Differences in mean word length did not reach significance (β = −.26, SE = .13, *t* = −2.02, adjusted *p* = .18) but showed a small‐to‐moderate effect size (Cohen's *d* = −.29). Effect sizes for the remaining non‐significant measures were small, ranging from Cohen's *d* = −.06 to .19.

**FIGURE 2:**
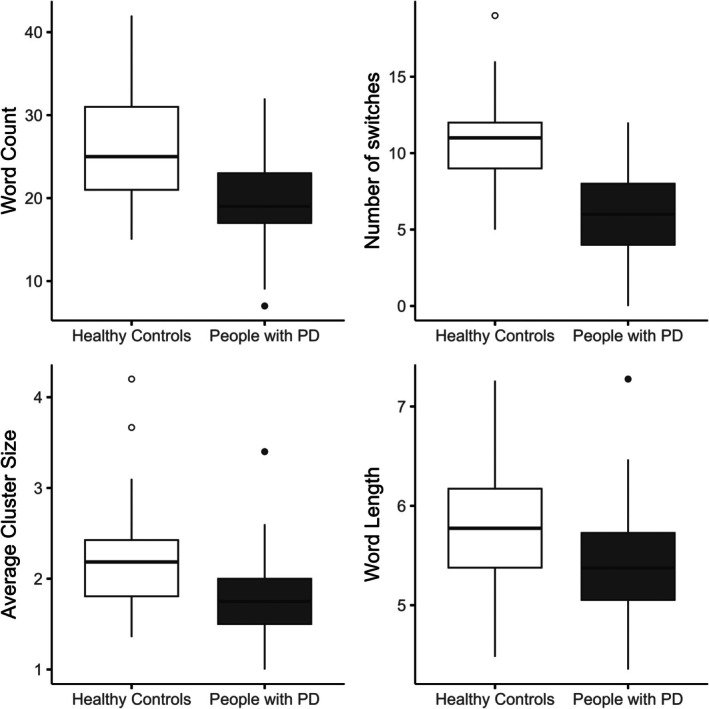
Significant differences between PD and healthy controls after DBS surgery. DBS, deep brain stimulation; PD, Parkinson's disease.

After surgery, people with PD produced significantly fewer words than healthy controls (β = −5.81, SE = 1.29, *t* = −4.55, adjusted *p* = .011; see Figure 2). This effect was large in size (Cohen's *d* = −.64). Similarly, patients made significantly fewer switches than controls (β = −4.52, SE = .60, *t* = −7.72, adjusted *p* < .001; Cohen's *d* = −1.09), representing a very large effect size. Additionally, PD patients showed significantly smaller average cluster sizes (β = −.36, SE = .12, *t* = −2.94, adjusted *p* = .019; Cohen's *d* = −.41) and produced significantly shorter words as measured by mean word length (β = −.44, SE = .15, *t* = −2.96, adjusted *p* = .019; Cohen's *d* = −.42). No significant differences were found between groups in mean age of acquisition (adjusted *p* = .63), mean word concreteness (adjusted *p* = .56), mean orthographic neighbourhood density (adjusted *p* = .30), mean phonological neighbourhood density (adjusted *p* = .23) or mean word frequency (adjusted *p* = .62). Effect sizes for these non‐significant variables were small (Cohen's *d* ranged from −.08 to .22).

### PD before versus after DBS surgery (Objective 2)

**FIGURE 3:**
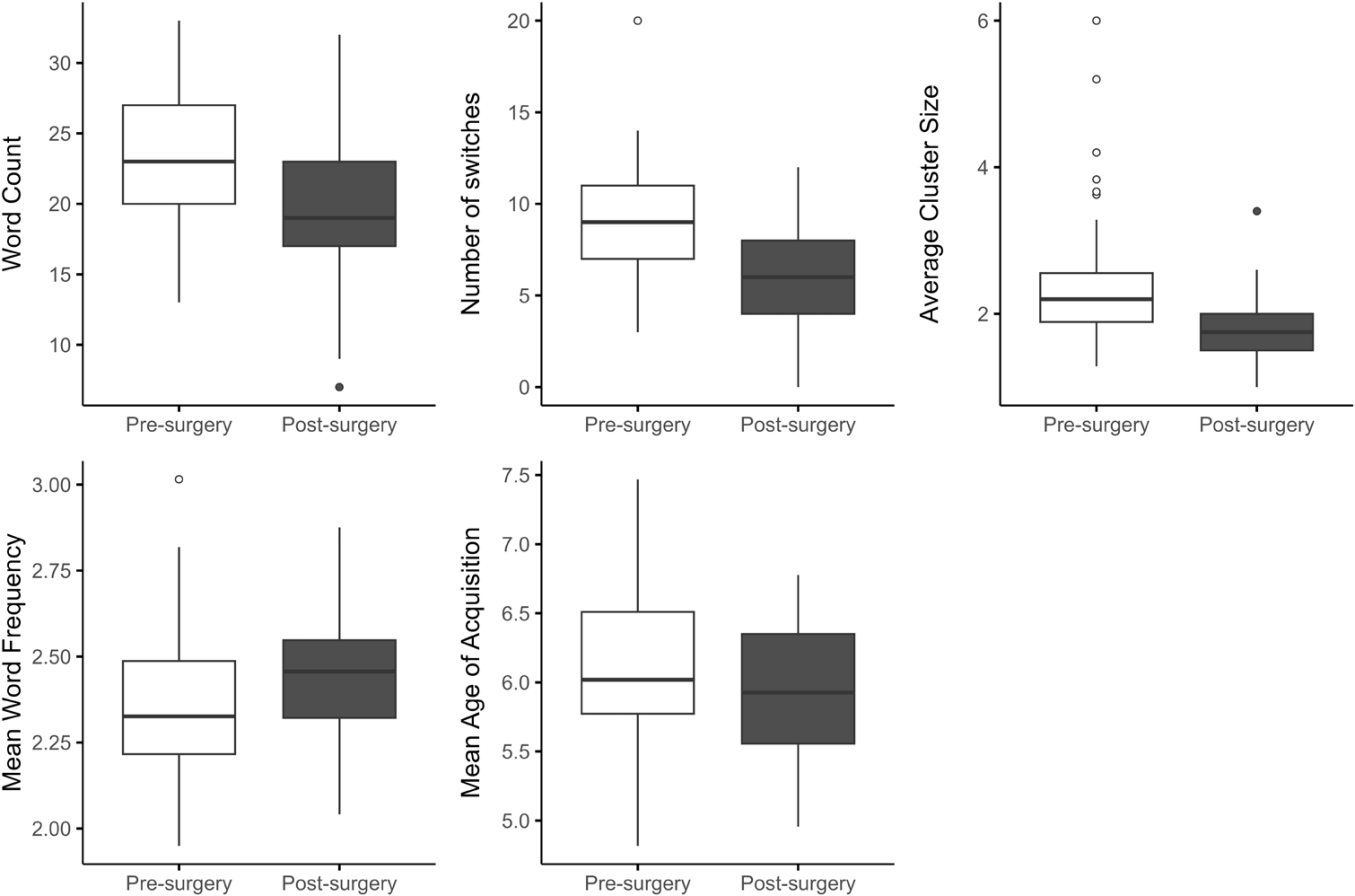
Significant differences in PD before versus after DBS surgery. DBS, deep brain stimulation; PD, Parkinson's disease.

Patients produced significantly fewer words after surgery compared to before surgery (β = −2.33, SE = .74, *t* = −3.16, adjusted *p* = .007; Cohen's *d* = −.39; see Figure 3). The number of switches after surgery was also significantly lower than before surgery (β = −3.09, SE = .43, *t* = −7.24, adjusted *p* < .001; Cohen's *d* = −.95; see Figure 3). The average cluster size was smaller post‐surgery than before surgery (β = −.50, SE = .12, *t* = −4.07, adjusted *p* < .001; Cohen's *d* = −.52; see Figure 3).

In addition, patients used significantly more frequent words after surgery compared to before (β = .06, SE = .03, *t* = 2.37, adjusted *p* = .047; Cohen's *d* = .34) and produced words acquired at an earlier age (β = −.16, SE = .07, *t* = −2.47, adjusted *p* = .047; Cohen's *d* = −.36; see Figure 3). The remaining item‐based factors did not reach statistical significance: concreteness (adjusted *p* = .86), mean orthographic neighbourhood density (adjusted *p* = .28), mean phonological neighbourhood density (adjusted *p* = .30) or mean word length (adjusted *p* = .35). Effect sizes for these variables were small, ranging from Cohen's *d* = −.01 to .18.

### Factors predicting total word count in PD differently than in healthy controls (Objective 3)

Before surgery, average cluster size was the only significant predictor of total word count in animal fluency, with a negative effect (β = −4.46, SE = 1.44, *t* = −3.09, adjusted *p* = .024). None of the other item‐based linguistic measures showed statistically significant predictive effects after correction for multiple comparisons: age of acquisition (β = −2.41, SE = 1.81, *t* = −1.34, adjusted *p* = .582), mean concreteness (β = 12.9, SE = 19.0, *t* = .68, adjusted *p* = .602), mean word length (β = .95, SE = 1.60, *t* = .59, adjusted *p* = .602), mean orthographic neighbourhood (β = .61, SE = .70, *t* = .88, adjusted *p* = .602), mean phonological neighbourhood (β = .32, SE = .47, *t* = .70, adjusted *p* = .602), mean word frequency (β = 7.22, SE = 4.75, *t* = 1.54, adjusted *p* = .582) and number of switches (β = −.33, SE = .34, *t* = −.98, adjusted *p* = .602).

**FIGURE 4:**
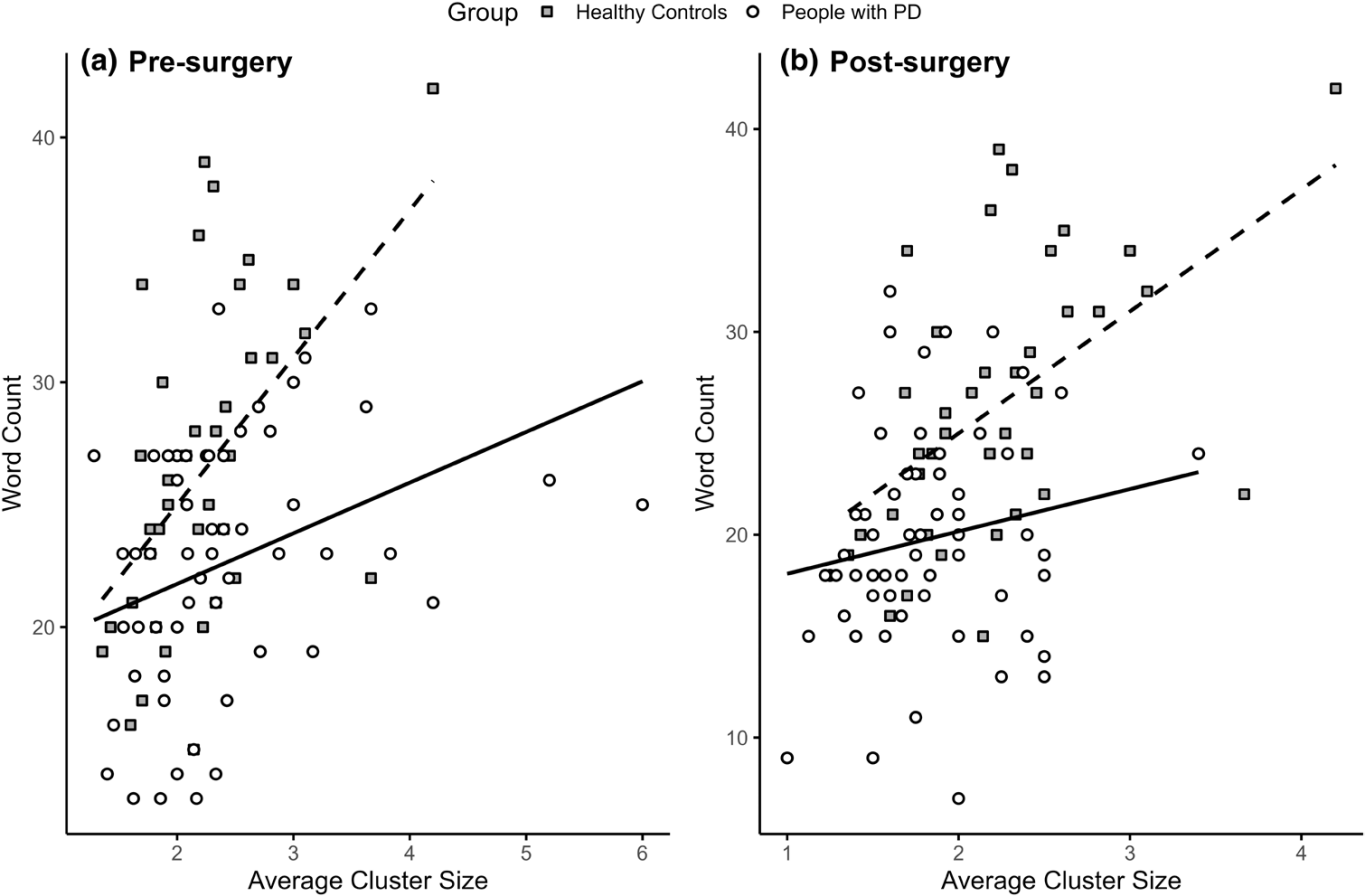
Interactions between group (healthy, PD) and average cluster size. To the left (4a) we present the PD data pre‐surgery (significant difference) and to the right (4b) PD data post‐surgery (not significant). PD, Parkinson's disease.

After surgery, none of the item‐based linguistic measures significantly predicted total word count in animal fluency for PD patients compared to healthy controls after correction for multiple comparisons: average cluster size (β = −3.07, SE = 2.06, *t* = −1.51, adjusted *p* = .690); age of acquisition (β = −2.42, SE = 2.11, *t* = −1.16, adjusted *p* = .690); concreteness (β = 11.8, SE = 20.6, *t* = .573, adjusted *p* = .720); word length (β = 1.65, SE = 1.68, *t* = .977, adjusted *p* = .690); orthographic neighbourhood density (β = 1.04, SE = .769, *t* = 1.36, adjusted *p* = .690); phonological neighbourhood density (β = .25, SE = .512, *t* = .490, adjusted *p* = .720); word frequency (β = 4.04, SE = 5.72, *t* = .714, adjusted *p* = .720); and number of switches (β = .127, SE = .348, *t* = .357, adjusted *p* = .723) (Figure 4).

### Correlation between factors predicting total word count and neuropsychological tests (Objective 4)

**FIGURE 5:**
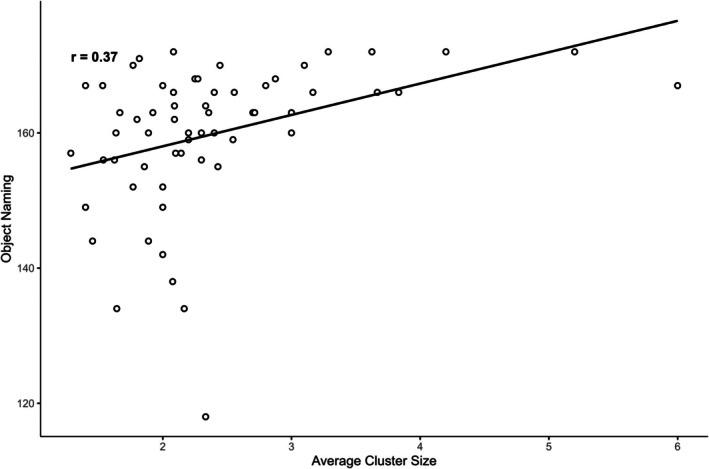
Correlation between average cluster size and object naming (pre‐operative data).

Before STN‐DBS surgery, we found significant positive correlations between average cluster size and the Boston Naming test (*r* = .37, *p* = .013, Bonferroni‐corrected; see Figure 5). No other significant correlations were observed between average cluster size and TMT Part B/A (*r* = −.13, *p* = 1.00, Bonferroni‐corrected), Stroop Card III/II (*r* = −.09, *p* = 1.00, Bonferroni‐corrected) or Digit Span Backward (*r* = .13, *p* = 1.00, Bonferroni‐corrected). All *p*‐values were adjusted using Bonferroni correction for multiple comparisons.

### Post‐hoc comparisons

A post‐hoc comparison of post‐operative scores (Wilcoxon signed‐rank test) showed no significant differences for the Boston Naming test (*V* = 843, *p* = .7164) and the Trial Making Test Part B/A (*V* = 803.5, *p* = .4138) Stroop card III/II test (*V* = 838, *p* = .4422).

## DISCUSSION

In this paper we set out to understand the mechanisms of decline in animal fluency by revealing impairments in language and/or executive functions in people with PD treated with STN‐DBS. Different from previous work, we studied item‐based characteristics of animal fluency, along with other more traditionally used fluency measures (i.e., total number of words, number of switches, cluster size). We compared the total number of correct words produced and item‐based factors (e.g., word frequency, number of switches, average cluster size) of the correct words produced between people with PD (before and after STN‐DBS surgery) and a group of healthy people (assessed one time) (Objective 1). We also studied whether the total number of correct words and the item‐based factors of fluency tasks differ within people with PD before and after STN‐DBS surgery (Objective 2). Moreover, we studied which item‐based factors (if any) predicted total word count in animal fluency in people with PD differently than in healthy controls before and after surgery (Objective 3). Finally, we studied whether the factors that predict total word count in animal fluency relate to tests of executive functioning and language processing (Objective 4). We accounted for factors argued to affect performance, namely, cognitive status, motor symptoms, disease duration and medication (Kübler‐Weller et al., 2025; Tröster, 2024).

Regarding Objective 1, before surgery, people with PD produced significantly fewer words and made fewer switches compared to healthy controls. After surgery, the differences in word count and number of switches became more pronounced, with larger effect sizes. New to the literature, people with PD produced significantly shorter words and had smaller average cluster sizes post‐surgery than healthy controls. To interpret these results, we will argue that the general pattern indicates that people with PD do not significantly differ from healthy controls on any of the item‐based measures before surgery. However, there is a general worsening of verbal fluency performance, which accentuates following STN‐DBS.

These results align with our predictions and have also been reported in previous research, particularly regarding the production of fewer words and fewer switches (Galtier et al., 2017; Henry & Crawford, 2004; Moretti et al., 2003; Mulders et al., 2021; Troyer et al., 1998). Producing fewer switches is common in individuals that produce fewer words (Rofes et al., 2023) and indicates difficulties in executive functions and particularly information updating and monitoring (Miyake et al., 2000). In both healthy people and people with neurological disorders, switches may occur when it is no longer possible to find words that belong to a subcategory (e.g., when they can no longer produce farm animals, they may start producing African animals). Therefore, for every switch, participants renew the criterium to find words, while keeping track of the words produced and the task instructions (Rofes et al., 2020, 2023). In the current study, the argument for difficulties in executive functions may be stronger before than after surgery, since no item‐based measures were affected before surgery. After surgery, having found shorter words and smaller average cluster sizes being significantly different between patients and controls, could be explained by issues with language and memory processes as well. In fact, shorter words have fewer phonemes, fewer syllables and simpler consonant clusters than longer words. Hence, shorter words typically require fewer phonological working memory resources and a lesser engagement of articulation than longer words (Whitworth et al., 2014). Also, producing smaller clusters may indicate difficulties retrieving lexical items and/or accessing specific concepts within the category animals (Busteed et al., 2025). That said, if people with PD after STN‐DBS would have severe difficulties in language, we would have probably detected more differences for lexico‐semantic item‐based measures between people with PD and controls (e.g., frequency, age of acquisition, concreteness). In fact, other authors have reported differences in age of acquisition between healthy people and people with PD (Wagner et al., 2020). However, we did not find such results for age of acquisition, when comparing people with PD against healthy controls.

Regarding Objective 2, the results show a significant decline in verbal fluency after STN‐DBS surgery (fewer words produced, fewer switches and smaller cluster sizes). These findings align with the comparisons between people with PD and healthy controls (i.e., Objective 1) as well as the aforementioned literature (e.g., Galtier et al., 2017; Henry & Crawford, 2004; Moretti et al., 2003; Mulders et al., 2021; Troyer et al., 1998). Interestingly, people with PD used more frequent and earlier‐acquired words after surgery, partially supporting the prediction of ‘easier’ word production post‐surgery. The post‐operative pattern could indicate a compensatory strategy or a shift towards more accessible lexical items as the disease progresses or as a result of the surgical intervention. The differences for frequency in animal fluency are in contrast with two other studies (Herrera et al., 2012; Zabberoni et al., 2017). However, previous work did not consider people with PD before and after STN‐DBS surgery but rather people with PD who had not been operated. Furthermore, frequency effects are not uncommon since they have been reported in other studies looking at other fluency tasks such as letter fluency (Foster et al., 2008), vegetable fluency (Tiedt et al., 2022) and action fluency (Herrera et al., 2012). Differences in age of acquisition like we found have also been reported in previous work (Wagner et al., 2020). Furthermore, from a statistical perspective it seems obvious to find effects for both frequency and age of acquisition since in our dataset, as well as in other studies, these two variables have been shown to correlate (Brysbaert & Ghyselinck, 2006).

Regarding Objective 3, before surgery, only average cluster size emerged as a significant predictor of total word count, whereby people with PD that produced more words also produced clusters that were bigger. After surgery, none of the item‐based measures showed statistically significant differences in predicting total word count. These findings suggest that the relationship between item‐based measures and overall fluency performance may be more complex than initially predicted. The lack of significant predictors could indicate that other factors not measured in this study might be more influential in determining verbal fluency performance in people with PD after STN‐DBS surgery. This point is stressed by the post‐hoc comparison of post‐operative scores that showed no significant differences for our measure of language (i.e., Boston Naming test) and two measures of executive functions: the Trial Making Test Part B/A for cognitive flexibility, and the Stroop card III/II for inhibition. Given these results, we could speculate for different factors beyond language that may play a role. Working memory, for example, has been related to producing shorter sentences during spontaneous speech (Batens et al., 2014, 2015). In fluency tasks, working memory is relevant for remembering the task instructions and keeping track of previous responses. Also, total number of words have been shown to correlate with Digit span scores in people with Mild Cognitive Impairment (Oh et al., 2019). Unfortunately, in our dataset we could not run perioperative comparisons for working memory, due to a lack of post‐operative scores for the Digit span backward.

The results of Objective 4 are therefore relevant to explain the patterns revealed in the previous analyses. In this regard, before surgery, we found a correlation between average cluster size and object naming (i.e., Boston Naming task), indicating that people producing larger average cluster sizes also score higher on object naming. These results align with previous results showing that cluster size is related to speed of lexical retrieval during object naming in people with PD (Fong et al., 2020; Gordon et al., 2018) as well as to fewer impairments with verbal fluency after STN‐DBS (Busteed et al., 2025). Since we did not find a correlation between average cluster size and any of the pre‐surgery measures (i.e., TMT Part B/A, Stroop Card III/II, Digit Span Backward), it could be the case that the total number of words is explained by language processing, particularly lexical retrieval, rather than executive functions such as working memory, mental flexibility or inhibition. These results therefore may strengthen the notion that language abilities, albeit not leading to severe impairments, are relevant to explain the performance of people with PD undergoing STN‐DBS, particularly before surgery. Having said that, the point we raised should be taken with caution since other cognitive functions that may be involved in cluster size, such as short‐term memory (e.g., Digit Span Forward), selective attention (e.g., TMT A), categorisation and abstraction (e.g., WCST) and cognitive flexibility (e.g., Letter Fluency or Modified‐Five Point test), were not assessed. Unfortunately, we could not run analyses with these measures in our current dataset.

In summary, we showed that people with PD have difficulties in animal fluency before and after STN‐DBS. New to the literature, item‐based measures of animal fluency may underscore language difficulties (e.g., post‐surgery, people with PD tend to use shorter words and have a smaller average cluster size compared to healthy individuals; they use more frequent and earlier acquired words after surgery compared to pre‐surgery; average cluster size predicts the total number of words before surgery and correlates with object naming). However, these difficulties might not be unique to language for different reasons: people with PD produced fewer switches after surgery, and even object naming also requires executive functions, for example, to search for the name of the object in the mental lexicon while inhibiting competing lexico‐semantic information (Whitworth et al., 2014). Finally, we interpret the finding that total number of words could not be predicted by any measures of fluency after surgery as an indication that other cognitive factors may be into play, when it comes to explain the performance of people with PD after STN‐DBS.

The results highlight the complex nature of verbal fluency deficits in PD, the role of executive functions and language abilities, and their worsening following STN‐DBS surgery. The findings indicate the importance of comprehensive pre‐ and post‐surgical neuropsychological assessments in people with PD undergoing STN‐DBS. In fact, these assessments helped us ratify the fact that language abilities play a role in our study. While, without this information, we would have been left with speculations based on other studies or the theoretical relations that can be established between item‐based measures and other parts of cognition (e.g., Whitworth et al., 2014). From a clinical perspective, the effect sizes reported for word count and number of switches highlight a need to consider the assessment of further language abilities in regular clinical protocols administered to this population. Adding to previous research, our results raise awareness of potential language impairments in this population, highlighting the need to assess lexico‐semantic processes, be it by analysing item‐based performance—like we do in this research by looking at word properties—and/or by considering new tasks or administering more consistently (e.g., other fluency tasks, naming).

### Limitations and future directions

One of the limitations of this study is that people with PD were assessed two times (i.e., before and after STN‐DBS surgery) while our control group was only assessed one time. The addition of a second assessment time for the control group would have been relevant to minimise potential test re‐test effects in the PD dataset. From a statistics point of view, adding this data would have allowed us to run an interaction model for Objective 1 (e.g., lmer(word_frequency~group*time + (1|participant), data = first_assessment)). Also, for Objective 3, adding a second assessment point for the control group would have allowed us to assess which pre‐operative variables in people with PD differently predict post‐operative number of words in people with PD versus controls. While addressing these questions is beyond the scope of the current study, they are important for future research. The same applies to comparing people with PD and STN‐DBS versus people without DBS to control for the progressive course of the disease and clearly elucidate effects of surgery. This type of control group, which has been used in several studies, includes people with PD under optimal medical treatment or people who decline the DBS procedure due to personal reasons (Avenali et al., 2025; Mikos et al., 2010). That said, 12 months is short enough to avoid a cognitive decline due to disease progression (Bucur & Papagno, 2023), though it is ethically too long for waiting list control groups, and even when control groups with PD and no surgery are used, effects of verbal fluency are also reported (Jahanshahi et al., 2022; Tröster, 2024).

Another limitation has to do with the number of participants in each of the groups. A retrospective power analysis showed that the number of participants entered in the analyses for total number of correct words and for number of switches was sufficient. However, the power obtained retrospectively for comparisons of other word properties was below 80%. This means that our results for word properties should be interpreted with caution and await replication with larger sample sizes.

In addition, while we controlled for some co‐founding factors regarding cognitive status, motor issues, medication, onset side of symptoms and disease duration, we could not control for variance in handedness, stimulation parameters, motivation, fatigue or learning effects, other than by indicating potential individual differences in our models. Evidence for effects of these factors is under scrutiny (Kübler‐Weller et al., 2025; Tröster, 2024) and could be considered in future work. In addition, PD is a neurodegenerative disease and even though we controlled for disease duration, there is a ‘time’ component between assessments that may affect the results beyond the implantation of the DBS system and the surgery themselves.

Future research could focus on investigating long‐term trajectory of animal fluency changes at different stages after STN‐DBS surgery, similar to other studies (Contarino et al., 2007; Jost et al., 2024). Longitudinal studies may be relevant to assess whether the findings we obtained can be replicated at any other stage after STN‐DBS surgery. Furthermore STN‐DBS settings such as stimulation frequency may influence the results (Busteed et al., 2025) and should be subject of future research. Fluency tasks targeting verbs may be used, given that people with PD tend to have problems with verb processing (Anyfantis et al., 2024; Colman et al., 2009). Finally, measures of fluency tasks could be explored, such as how words may group from the perspective of word networks and graph theory (Arias‐Trejo et al., 2021; Zhang et al., 2024).

## CONCLUSION

People with PD before and after STN‐DBS surgery produce fewer words than healthy controls in animal fluency. Item‐based characteristics extracted from animal fluency indicate difficulties that can be attributed to executive functions and language processing. Our findings agree with the current literature, adding some indication that lexical retrieval may be one of the key factors explaining the total number of words before surgery. Different linguistic or cognitive factors may be affecting performance after surgery. Replication with further participants is needed to ratify these results.

## AUTHOR CONTRIBUTIONS

**Adrià Rofes:** Conceptualization; methodology; investigation; writing – original draft; writing – review and editing; data curation; supervision; visualization; project administration. **Nikki Janssen:** Methodology; writing – review and editing; writing – original draft; supervision; data curation; project administration. **Janine Rook:** Writing – original draft; methodology; writing – review and editing; data curation; project administration. **Eva de Ronde:** Writing – review and editing; project administration; supervision; methodology. **R. Saman Vinke:** Writing – review and editing; project administration; data curation; methodology. **Rianne A. J. Esselink:** Writing – review and editing; project administration; methodology. **Annelien A. Duits:** Writing – original draft; conceptualization; funding acquisition; methodology; writing – review and editing; project administration; data curation; supervision; resources.

## CONFLICT OF INTEREST STATEMENT

The authors declare no conflicts of interest.

## Data Availability

The data that support the findings of this study are available from the corresponding author upon reasonable request.
